# Dune Hares: Population Indices, Home Range Size, and Habitat Selection of the European Hare on a Danish Island

**DOI:** 10.1002/ece3.70415

**Published:** 2024-10-08

**Authors:** Majbrit Högberg Kleist, Rasmus Mohr Mortensen, Thomas Bregnballe, Martin Mayer

**Affiliations:** ^1^ Department of Ecoscience Aarhus University Aarhus Denmark; ^2^ Department of Forestry and Wildlife Management Inland Norway University of Applied Sciences Koppang Norway

**Keywords:** Langli island, *Lepus europaeus*, mammal, spatial ecology, Wadden Sea

## Abstract

Population indices, such as transect counts of animals, can provide important information concerning population changes over time. Moreover, data concerning the home range size and habitat selection of individuals can provide valuable insight into spatial requirements of animals and how they can adapt to variable environments. Here, we describe the population development of European hares (*Lepus europaeus*) and investigated home range sizes and habitat selection of six radio‐tagged individuals on the small (80 ha) Danish Wadden Sea island Langli. The average minimum hare population density from 1983 to 1997 was 64 ± 36 (mean ± SD) hares/km^2^, with hare numbers varying among years and seasons. The average home range size was 23.3 (CI: 18.9–28.7) ha, which is comparable to agricultural areas of high structural diversity. Moreover, hare habitat selection was generally bimodal, with hares moving over larger areas and selecting marsh habitat for foraging during nighttime, and dune and grassland habitat for resting during daytime, especially during winter. Combined, our results indicate that hare abundance and space use in the dunal landscape of Langli Island were similar to agricultural areas of comparatively high habitat quality. Thus, dunal marsh landscapes offer high‐quality habitat for hares and might be of importance as population strongholds at a time when hare populations are declining in many agricultural areas across Europe.

## Graphical abstract

We investigated the home range sizes and habitat selection of six radio‐tagged European hares on the small Danish Wadden Sea island Langli. Our results indicate that hare space use in the dunal landscape of Langli Island was similar to agricultural areas of comparatively high habitat quality. Thus, dunal marsh landscapes offer high‐quality habitat for hares, and might be of importance as population strongholds.

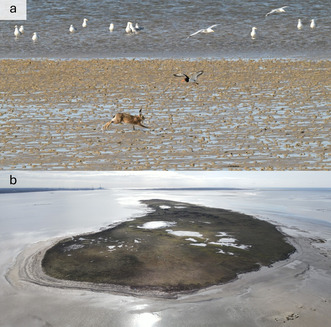

## 1 Introduction

Population monitoring is a crucial tool in ecology, allowing us to track relative or absolute changes in the number of individuals in a population over time. Generally, population density estimates are positively correlated with measures of habitat availability and quality (Johnson et al. 2006; Burger and Waterhouse 2009). Moreover, understanding habitat selection and home range sizes are important topics in wildlife ecology and conservation, because these factors provide valuable information concerning the spatial ecology of animals (Raynor et al. 2017; Waterman et al. 2020). Home range size is often used as indicator of habitat quality and connectivity, with smaller home ranges indicating higher resource availability (Walter et al. 2018). Furthermore, the study of habitat selection can reveal the flexibility of animals in response to environmental variation (Mayer et al. 2019; Mortensen et al. 2022). Special habitats, such as small dunal islands, can provide unique insights into this adaptability.

**FIGURE 1:**
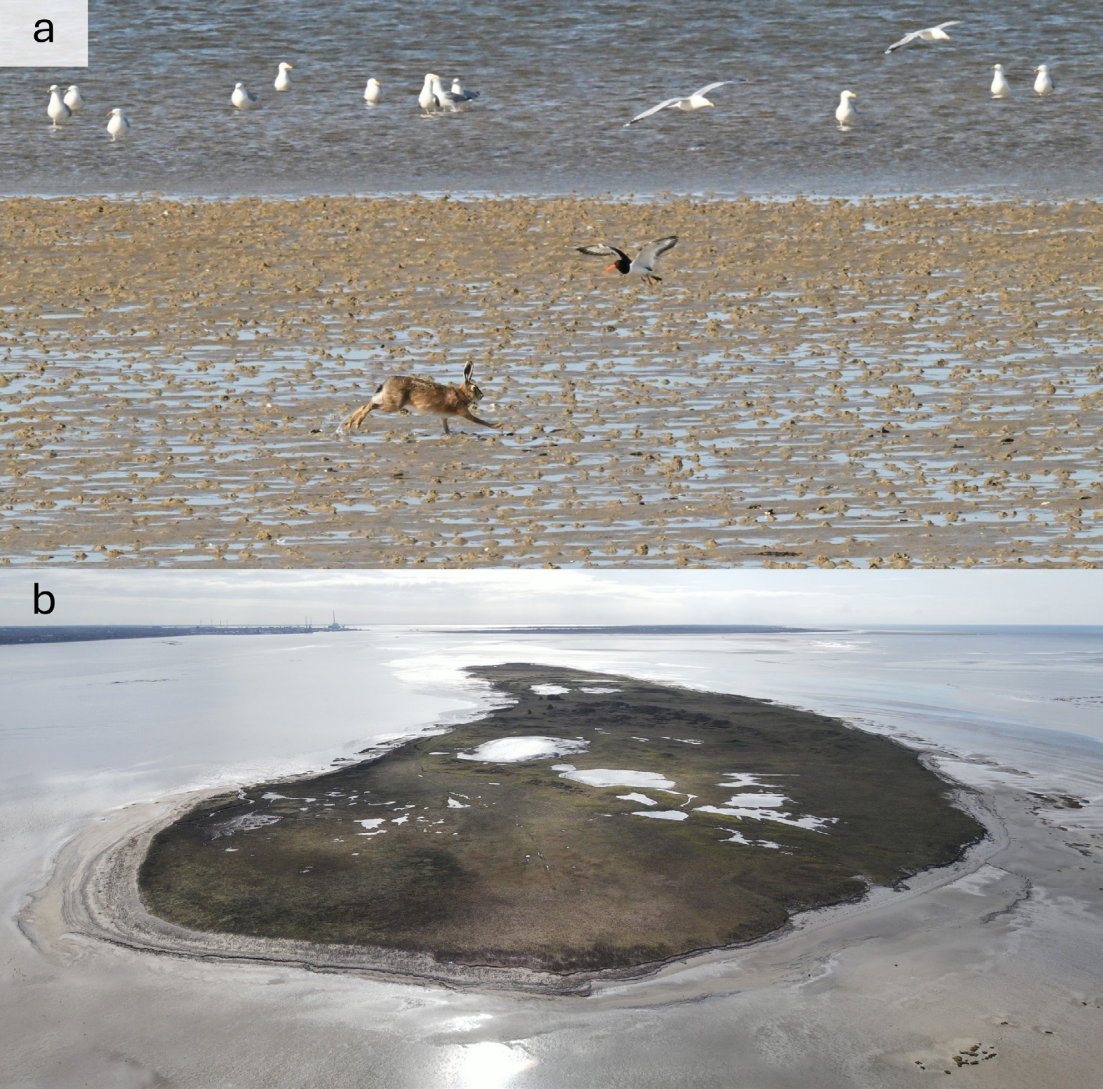
A European hare (*Lepus europaeus*) on Langli Island (a), and an aerial image of Langli Island (b). Pictures: Lars Maltha Rasmussen.

The European hare (*Lepus europaeus*, hereafter hare, Figure 1) is a medium‐sized, non‐territorial mammal that mostly lives in open landscapes. It is an important species for hunting and has been introduced in several countries outside its native distribution (Stott 2015; De Faria et al. 2016). Agricultural land is now the main habitat of the hare, with populations in Europe having experienced population declines since the 1960s, mostly because of agricultural intensification leading to the reduction of high‐quality year‐round forage and cover (Frylestam 1986; Vaughan et al. 2003; Smith, Jennings, and Harris 2005; Mayer et al. 2019). Several studies have estimated home range size and habitat selection of hares in agricultural landscapes (Schai‐Braun and Hackländer 2013; Schai‐Braun et al. 2014; Mayer et al. 2018, 2022; Levänen, Pohjoismäki, and Kunnasranta 2019). However, less is known about home range size and space use of hares in other habitat types, such as urban areas (Mayer and Sunde 2020a; Bach, Escoubet, and Mayer 2023) and salt marshes (Kunst, Van der Wal, and van Wieren 2001). Here, we describe population estimates, home range sizes, and habitat selection of hares in a dunal habitat, located on an island in the Danish Wadden Sea, and discuss how they compare to densities and space use of hares in agricultural landscapes.

## 2 Material and Methods

### 2.1 Study Area and Habitat

**FIGURE 2:**
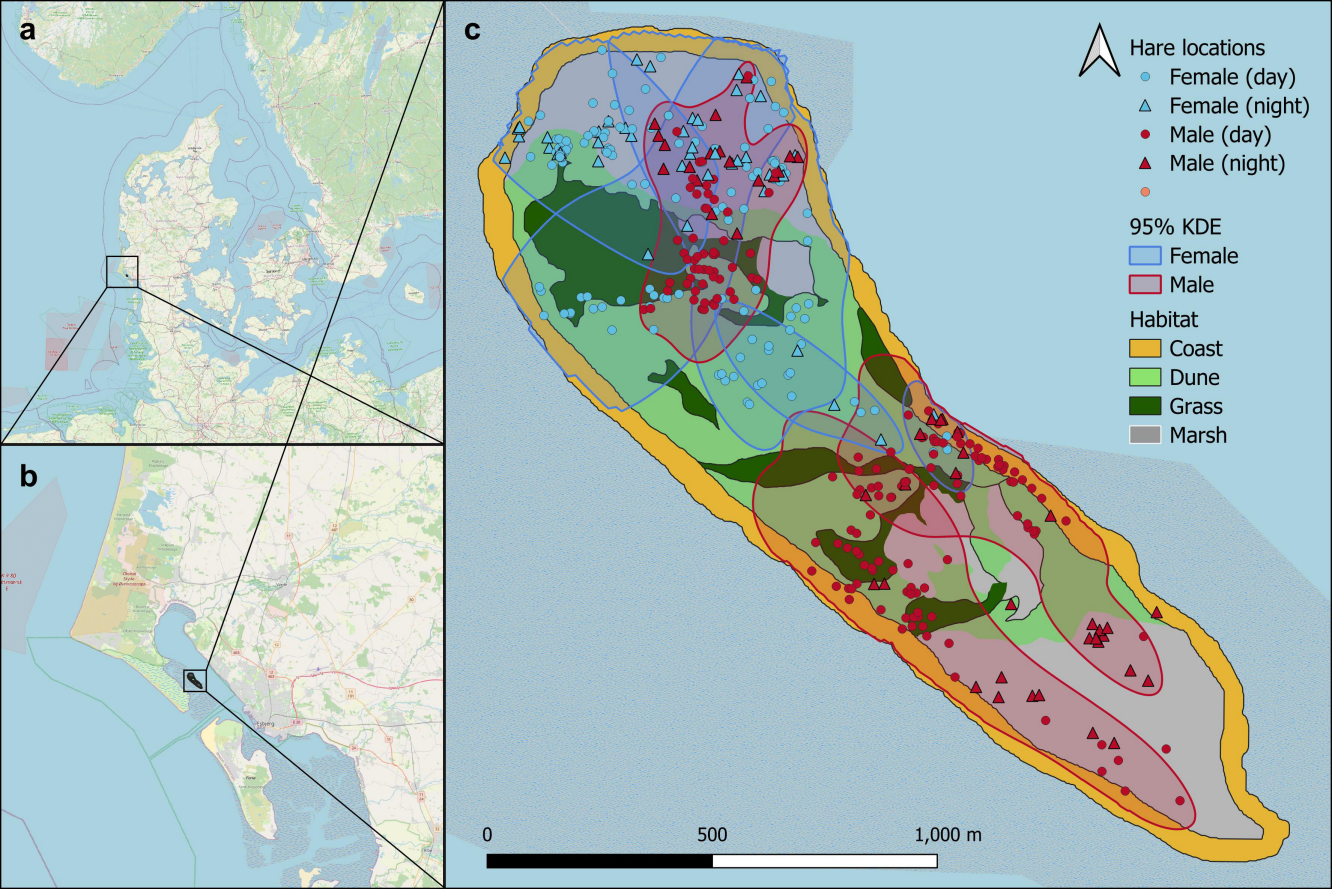
Overview of the location of our study area, the island of Langli, within Denmark (a, b), and showing the different habitat types, European hare (*Lepus europaeus*) locations, and 95% kernel density estimates (KDE) separately for females and males (c).

Langli is a Danish Wadden Sea island situated in Ho Bay (55.0 30′N, 08.0 20′E; Figure 2), the northernmost part of the Wadden Sea. The island is 80 ha in size and dominated by a dunal landscape, which covers the middle part of the island. Larger areas of salt marsh, which are regularly flooded, cover the north and south parts of the island. The soil is mostly sandy, and the vegetation is dominated by different salt‐tolerant grass species.

Habitats were categorized into four categories based on aerial pictures and ground surveys to monitor vegetation. (1) Grass, defined as flat field‐like areas with comparatively tall ground vegetation, rarely covered by salt water; dominated by grasses, primarily *Festuca rubra*, *Elymus repens*, and *Poa* spp. This habitat covered 17% of Langli (13.6 ha). (2) Dune, defined as hilly, dry dunal landscape with comparatively tall ground vegetation and free of any saltwater coverage throughout the year; dominated primarily by *Deschampsia flexuosa*, *Agrostis capillaris*, and *Carex arendria*, interspersed with *Empetrum nigrum*, *Calluna vulgaris*, and *Salix repens*. This habitat covered 40% of Langli (32 ha). (3) Coast, defined as flat, sandy areas with short vegetation. The lowest part of the coast is flooded during some of the spring tides; vegetation mostly consists of *Festuca rubra*, *Puccinellia maritima*, *Seriphidium maritimum*, *Juncus gerardii*, *Limonium vulgare*, and *Glaux maritima*. This habitat covered 17% of Langli (13.6 ha). (4) Marsh, defined as flat areas regularly influenced by salt water with short vegetation; vegetation dominated by *Festuca rubra*, *Puccinellia maritima*, *Juncus gerardii*, *Seriphidium maritimum*, *Glaux maritima*, *Limonium vulgare*, *Atriplex prostrata*, and *Triglochin maritima*. This habitat covered 26% of Langli (20.8 ha).

Red foxes (*Vulpes vulpes*) that tried to settle on the island in winter were regulated by hunting, and there were few other hare predators; European herring gulls (*Larus argentatus*) and hooded crows (*Corvus cornix*) have been observed attacking leverets (unpublished data). There was no agriculture and no road infrastructure on the island. The only humans present on the island during the study period were the one to three people working at the islands' ornithological field station.

### 2.2 Hare Population Monitoring

To estimate hare numbers and monitor relative changes in the hare population from 1983 to 1997, we obtained data from transects that were walked by professional ornithologists during daytime who recorded hares while counting Eurasian skylark (*Alauda arvensis*) and meadow pipit (*Anthus pratensis*). Transects were 4.26 km in length and crisscrossed the island, allowing visual surveying of the entire island area. Counts were conducted 4–45 times (mean ± SD: 29 ± 7) per season (spring: March to May, summer: June to August, fall: September to November, and winter: December to February) each year. We used the maximum number of hares observed during a single transect survey as a minimum hare population estimate for each season and year, noting that this number is an underestimate as not all hares are active and visible during daytime. To obtain more reliable population estimates, MHK walked the same transects between 9 and 11 pm from March 1990 to February 1991, conducting 11–12 surveys per season. A Maglite flashlight (ML150LR‐4019 MAGLITE ML150LR with up to 1082 lumen) was used to detect hares. We here assume that MHK recorded most hares present and that each hare was recorded only once. We again used the maximum number of hares recorded within each season as a minimum estimate of population size (noting that some hares likely were not detected).

### 2.3 Hare Captures and Tracking

In September 1990, three female and three male hares were caught by driving them into nets. Each hare was individually ear‐tagged with reflectors of different color combinations and equipped with a TXT‐1Sm necklace‐mounted radio transmitter of 50 g, weighing about 1%–1.3% of the hare's body weight (4000–4700 g). Two handheld antennas and a Radio receiver (AVM Instrument Co.nr. 12599) were used for tracking. Radio‐collared hares were located 5–10 days a month from September 1990 to April 1991 by homing in on the signal until we could observe the hare from a distance (10–100 m), recording their position on a paper map. A maximum of three radio fixes per day were obtained and no two fixes were closer than 3 h apart.

### 2.4 Data Preparation and Analysis

We analyzed the maximum number of hares observed (response variable) on daytime transects using generalized additive models of the R package “mgcv” with a binomial error distribution using a restricted maximum likelihood (REML) approach (Wood 2017). We included year as smooth term and season as parametric predictor variable in the model. Basis dimensions for the parameter k were checked with “gam.check” function (Wood 2017).

For the radio‐tracked hares, we estimated their total and seasonal (fall, winter, and spring) home range size based on all locations as 95% autocorrelated kernel density estimates (AKDE) (Fleming et al. 2015) using the R package “ctmm” (Calabrese, Fleming, and Gurarie 2016). Due to the sampling frequency, all movement models assumed independent and identically distributed sampled locations, which corresponds to conventional kernel density estimates (KDE). The proportion of overlapping home ranges between individuals and seasons was assessed by estimating the Bhattacharyya coefficient, an index ranging from 0 (no overlap) to 1 (total overlap) (Winner et al. 2018). Average home range sizes and proportional overlaps were calculated using generalized linear mixed models with individual hare ID as random effect and Gamma and beta error distributions, respectively, weighted by the degrees of freedom of the movement models.

Based on the hare locations obtained from radio‐tracking, we investigated hare habitat selection. To sample available habitat, we created five random locations for each hare observation, which were assigned the same date and time as the hare location. We then used generalized linear mixed models (GLMMs) with observation type (hare location = 1 versus random location = 0) as response variable and individual hare ID as random effect. We initially included the interaction of sex with habitat type as fixed effect but found no effect of sex and thus omitted this variable from the further analyses (also because our sample size was too small to make a valid comparison between females and males). Consequently, we included time of the day (defined as “day” between 08:00 and 19:00 and “night” between 19:01 and 07:59), habitat type, and the interactions of habitat type × time of the day as fixed effect. After detecting an effect of time of the day (see results) and to avoid higher‐order interactions, we ran separate analyses for day and night locations, including season (fall, winter, spring), habitat type, and their interaction as fixed effects. Model selection was based on AICc (Burnham, Anderson, and Huyvaert 2011) and was carried out using the R package MuMIn (Barton 2016). Parameters that included zero within their 95% CI were considered uninformative (Arnold 2010). We validated the most parsimonious models by plotting the model residuals versus the fitted values (Zuur and Ieno 2016). All statistical analyses were carried out in R 4.3.3 (R Core Team 2024).

## 3 Results

**FIGURE 3:**
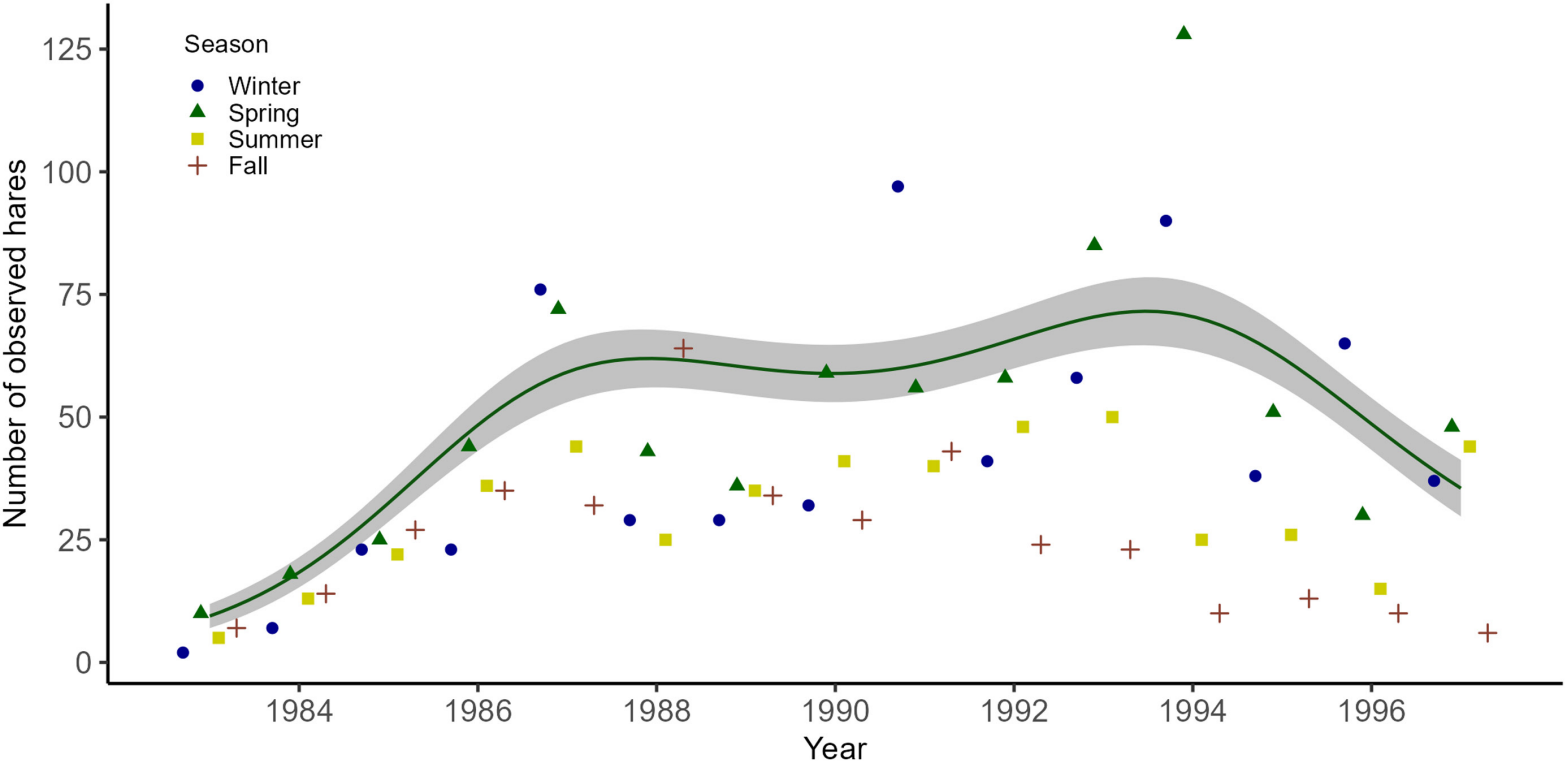
The maximum number of hares observed during diurnal transect surveys in the years 1983–1997, shown separately for each season (small symbols). The line shows the effect of year on the predicted number of observed hares in spring. The 95% confidence interval is shown as shading.

Based on diurnal surveys, the number of observed hares increased from 1983 to 1986, was stable from 1987 to 1994, and then decreased from 1995 to 1997 (Figure 3, Table 1). During these three periods, spring count numbers corresponded to a minimum hare density (mean ± SD) of 30 ± 18 hares/km^2^ in 1983–1986, 84 ± 36 hares/km^2^ in 1987–1994, and 54 ± 14 hares/km^2^ in 1995–1997. There was considerable variation in the number of counted hares among seasons, also within years, with fall counts generally being lowest (Figure 3, Table 1). The estimated hare population density based on nocturnal transect counts from March 1990 to February 1991 was 118 hares/km^2^ (spring), 99 hares/km^2^ (summer), 188 hares/km^2^ (fall), and 126 hares/km^2^ (winter). Transect counts conducted during nighttime resulted in on average 2.5‐fold more hare observations compared to diurnal transect counts conducted in the same season.

**TABLE 1:** Model estimates for parametric coefficients and smooth terms of the most parsimonious generalized additive model investigating the number of hares observed during transect counts conducted from 1983 to 1997 on Langli Island. Winter was used as reference level.

Parametric coefficients	Estimate	SE	*z* value	*p*
Intercept	3.66	0.04	88.71	< 0.001
Season spring	0.16	0.05	2.94	0.003
Season summer	−0.34	0.06	−5.54	< 0.001
Season fall	−0.58	0.07	−8.84	< 0.001
Smooth terms	edf	Ref.df	Chi.sq	*p*
s(continuous month)	3.94	4.00	268.40	< 0.001

Abbreviations: Chi.sq. = chi squared, edf = estimated degrees of freedom, Ref.df = reference degrees of freedom, SE = standard error.

Between September 1990 and April 1991, we obtained 487 hare locations from 6 radio‐tracked hares. The average 95% KDE of the 6 hares was 23.3 ha (CI: 18.9–28.7) with an overlap coefficient of 0.28 (CI: 0.20–0.37) (Table 2, Figure 2). Female 95% KDEs were on average 27.4 ha (CI: 21.4–35.0) compared to 19.8 (CI: 15.8–24.5) ha in males (Table 2). Due to the small sample size, we could not quantify sex differences concerning home range size and overlap. No seasonal differences were found in individuals' 95% KDEs. The average 95% KDE was 16.4 (CI: 10.9–24.7) ha in fall, 22.1 (CI: 15.7–31.2) ha in the winter, and 18.1 (CI: 13.2–24.8) ha in the spring. Individuals' seasonal home ranges overlapped with a proportion of 0.64 (CI: 0.52–0.75). Night 95% KDEs were on average 32.6 (CI: 22.1–48.2) ha and considerably larger than day 95% KDEs that were 22.9 (CI: 16.8–31.2) ha. The proportion of overlap between day and night of each individual was 0.84 (CI: 0.22–0.99).

**TABLE 2:** Overview of the 6 radio‐collared hares, showing their sex, number of obtained locations, number of individual monitoring days, 95% kernel density estimates (KDE) as measure of home range size, and seasonal 95% KDEs.

Hare ID	Sex	Number of locations	Monitoring days	95% KDE (ha)	Fall 95% KDE (ha)	Winter 95% KDE (ha)	Spring 95% KDE (ha)
Blue	Male	66	39	27	13	20	24
Green	Female	133	42	33	15	22	32
Red	Female	41	20	26	20	23	
Red/yellow	Male	87	40	18	21	18	15
White	Male	76	44	15	11	18	14
Yellow	Female	84	42	23	19	31	5

**FIGURE 4:**
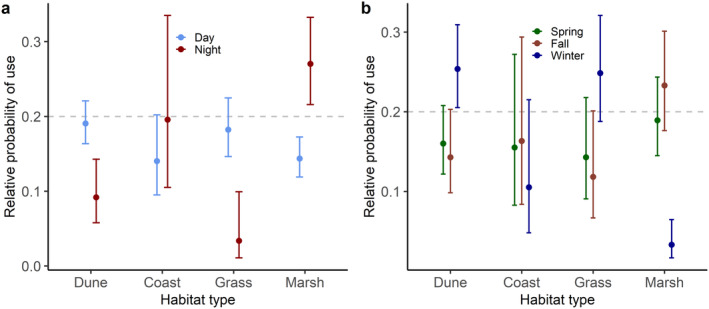
The predicted effect of (a) the interaction of habitat type × time of the day and (b) the interaction of habitat type × season for daytime locations on the relative probability of use by 6 European hares (*Lepus europaeus*) on Langli Island. Values > 0.2 indicate selection, whereas values < 0.2 indicate avoidance. The 95% confidence intervals are given as bars.

**FIGURE 5:**
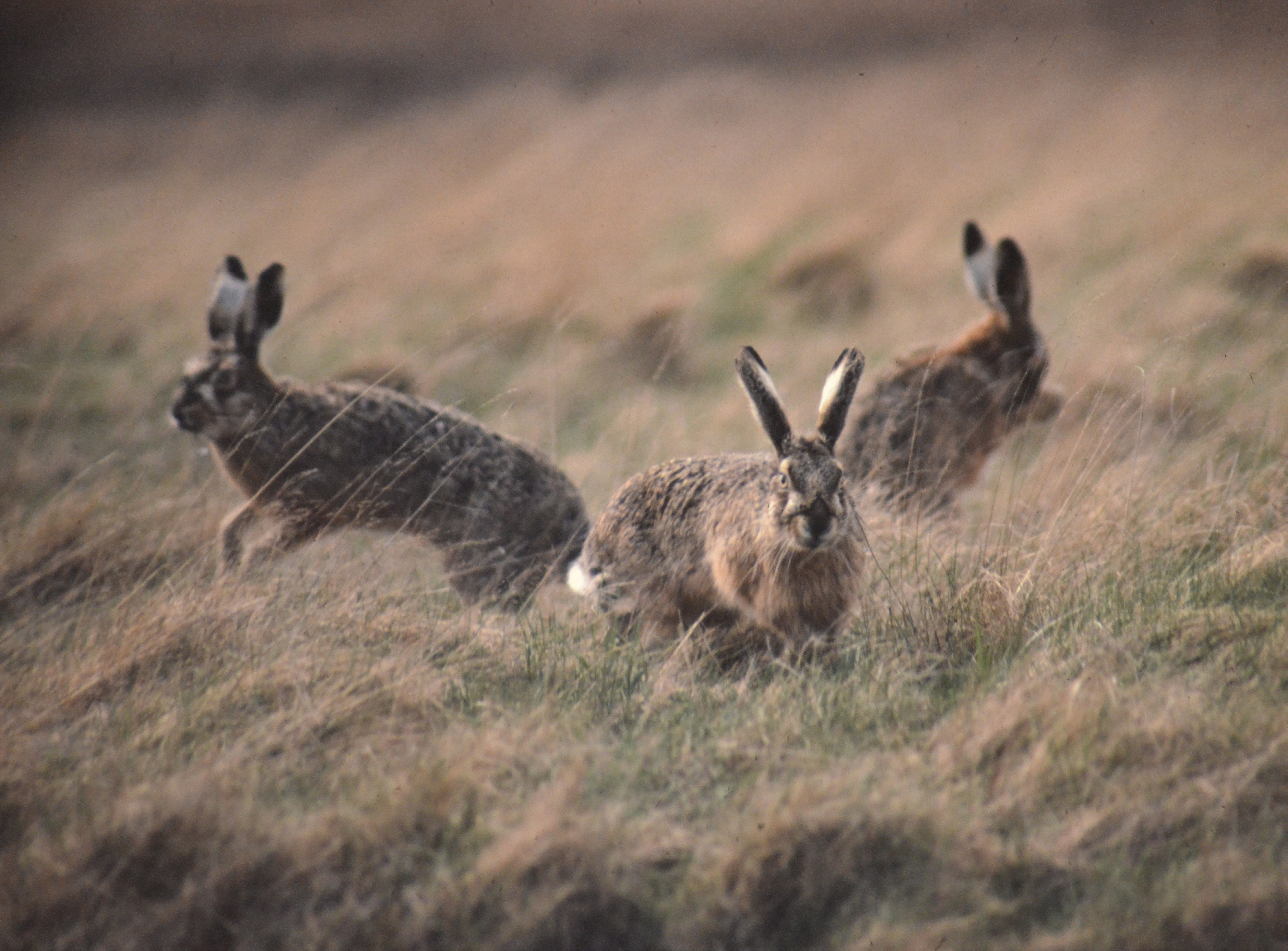
A group of three European hares (*Lepus europaeus*) observed on Langli island. Picture: Lars Maltha Rasmussen.

During daytime, hares avoided marsh habitat, tended to avoid coastal areas, and showed no clear selection or avoidance for dune and grassland habitat (Figure 4a, Table 3). Conversely, during nighttime, hares avoided dune and grassland habitat, selected for marsh habitat, and showed no clear selection or avoidance for coastal areas. During nighttime, we regularly observed 10–20 hares foraging in loose groups; however, we did not quantify grouping behavior further (Figure 5). When running separate analyses for night and day locations, we found no evidence that hare habitat selection during nighttime changed seasonally, because the interaction between season × habitat was not retained in the most parsimonious model (Table 3). During daytime, habitat selection by hares changed seasonally (Figure 4b, Table 3). During winter days, hares selected for dune habitat, avoided marshes, and showed no selection or avoidance of coast and grass habitat (Figure 4b). During spring and fall days, hares did not show a clear selection or avoidance of any habitat type but tended to avoid dune and grassland habitat (Figure 4b).

**TABLE 3:** Model estimates, standard error (SE), lower 95% confidence interval (LCI) and upper 95% confidence interval (UCI) of explanatory variables for the analyses of habitat selection by European hares on Langli, separately for (1) all hare locations, (2) locations obtained during nighttime, and (3) locations obtained during daytime. Informative parameters are given in bold. Positive estimates indicate a higher relative probability of use (selection), whereas negative values indicate a lower relative probability of use (avoidance). Daytime, fall season, and dune habitat were defined as reference categories.

Variable	Estimate	SE	LCI	UCI
(1) Habitat selection estimated from all hare locations				
Intercept	−1.45	0.09	−1.63	−1.26
Time of day night	**−0.84**	**0.27**	**−1.38**	**−0.31**
Habitat coast	−0.37	0.24	−0.85	0.11
Habitat grass	−0.05	0.16	−0.38	0.27
Habitat marsh	**−0.34**	**0.15**	**−0.62**	**−0.05**
Time of day night × Habitat coast	**1.24**	**0.51**	**0.24**	**2.25**
Time of day night × Habitat grass	−1.01	0.66	−2.31	0.29
Time of day night × Habitat marsh	**1.64**	**0.33**	**0.99**	**2.28**
(2) Habitat selection estimated from night locations				
Intercept	−2.29	0.25	−2.79	−1.79
Habitat coast	0.88	0.45	−0.01	1.76
Habitat grass	−1.07	0.64	−2.32	0.19
Habitat marsh	**1.30**	**0.30**	**0.72**	**1.88**
(3) Habitat selection estimated from day locations				
Intercept	−1.79	0.22	−2.22	−1.37
Season spring	0.13	0.27	−0.40	0.66
Season winter	**0.71**	**0.26**	**0.21**	**1.22**
Habitat coast	0.16	0.44	−0.71	1.03
Habitat grass	−0.22	0.39	−0.98	0.54
Habitat marsh	**0.60**	**0.28**	**0.05**	**1.15**
Season spring × Habitat coast	−0.20	0.60	−1.36	0.97
Season winter × Habitat coast	−1.22	0.63	−2.46	0.02
Season spring × Habitat grass	0.08	0.50	−0.89	1.05
Season winter × Habitat grass	0.19	0.45	−0.69	1.07
Season spring × Habitat marsh	−0.40	0.36	−1.11	0.31
Season winter × Habitat marsh	**−2.90**	**0.48**	**−3.83**	**−1.96**

## 4 Discussion

We provided minimum estimates of hare numbers and investigated home range sizes and habitat selection by six radio‐collared hares on the small island of Langli. Based on transect counts conducted during nighttime, the minimum hare population density in spring 1991 was 118 hares/km^2^. Compared to hare density estimates from agricultural areas, this estimate is at the high end, comparable to heterogeneous arable areas with various crops and fallow habitat (Bertóti 1975; Hackländer et al. 2001; Smith, Jennings, and Harris 2005), and is similar to estimates from the Danish island Illumø (Barndorff‐Nielsen, Abildgård, and Andersen 1972). The estimate is much higher compared to recent hare density estimates on the Danish mainland that ranged from 0.8 to 34.4 hares/km^2^ (Mayer and Sunde 2020b) and compared to other countries with agricultural areas dominated by pastural systems or monocultures (Smith, Jennings, and Harris 2005). Moreover, hare density estimates from transect counts conducted at night were ca. 2.5‐fold higher compared to day transect counts conducted in the same season and year. This suggests that the long‐term trend of the Langli hare population was likely at least double than presented here. Nevertheless, these indices provide a proxy of how the hare population has changed over time. Apart from biological effects, such as within‐year variation in reproductive output and survival, the large differences among seasons were probably related to differences in detection probability due to variation in vegetation height, as well as differences in hare activity.

To our knowledge, only one other study to date investigated space use by European hares in a natural coastal landscape, on a Dutch salt marsh island (Kunst, Van der Wal, and van Wieren 2001). Here, the average hare home range size (also based on six individuals) was 29 ha, which is comparable to the 23 ha for the hares on Langli. Similarly, home range sizes did not differ between sexes and across seasons (Kunst, Van der Wal, and van Wieren 2001). Compared to studies from agricultural areas (Broekhuizen and Maaskamp 1982; Tapper and Barnes 1986; Marboutin and Aebischer 1996; Rühe and Hohmann 2004; Ferretti et al. 2010; Mayer et al. 2018) that reported home range sizes between 21 and 101 ha based on 95% to 100% KDEs or minimum convex polygons (MCP), the home range size of this study is at the lower end. Given that home range size is a predictor for habitat quality, this suggests that the habitat quality in this dunal landscape was comparable to arable areas with high structural diversity, such as smaller agricultural field sizes and various crop types (Schai‐Braun and Hackländer 2013; Mayer et al. 2018). Moreover, distinct home ranges that did not cover the entire island suggest that the relatively small area of Langli is sufficient to support a hare population, also because the hare population persisted there for > 70 years (unpublished data). However, it is important to note that as hares in this study were tracked less frequently compared to studies utilizing GPS technology (Mori et al. 2022), the comparatively smaller home range sizes reported here might partly be owed to fewer relocations per individual. Moreover, our sample size of six hares was small, cautioning against too broad generalizations from these data alone.

Generally, home ranges were bimodal covering different areas during daytime for resting and during nighttime for foraging, in line with findings from previous studies (Tapper and Barnes 1986). During daytime, hares were more likely to be in dune and grass habitats (though they did not select for these areas compared to their availability), and during nighttime they selected for marsh habitat and were more likely to be in coast habitat (compared to daytime locations). The preference of marsh habitat and use of coast habitat by active hares was likely related to the availability of preferred plant species for foraging. Additionally, marsh habitat consisted of large, flat areas offering high visibility, making it possible for the hares to gather and keep in visual contact with each other during foraging. Thus, the selection for open habitat (Mayer, Fog Bjerre, and Sunde 2020) and clustering with conspecifics (Broekhuizen and Maaskamp 1982) may be an anti‐predation behavior. The avoidance of dune and grassland habitat during nighttime, when hares were foraging, can be explained by their comparatively high vegetation, as hares were previously shown to select for short vegetation when foraging (Mayer et al. 2018). Habitat selection based on nighttime locations did not differ seasonally. In contrast, during daytime, hares tended to select for dune and grassland habitat and avoided marshes during winter but showed no clear selection or avoidance for any habitat type during spring and fall. A possible reason for the avoidance of marsh during winter could be that the vegetation is comparatively short in this habitat, offering little refuge during the cold time of the year, with grassland and especially dune habitats offering more thermal shelter, due to their tall ground vegetation and because they are dry year‐round. Similarly, hares in agricultural areas selected for tall ground vegetation during winter, likely as shelter from cold conditions, but selected for comparatively shorter vegetation during the rest of the year (Mayer et al. 2019).

In summary, hare population density indices, home range sizes, and patterns of habitat selection in the dunal landscape of Langli Island were comparable to hare densities and space use in agricultural areas with high structural diversity, reflecting comparatively high habitat quality. Thus, dunal marsh landscapes offer high‐quality habitats for hares and might be of increasing importance as population strongholds at a time when hare populations are declining in many agricultural areas across Europe. Continued surveys on Langli Island would be valuable to understand if the hare population density has remained similarly high over the last two decades.

## Author Contributions

**Majbrit Högberg Kleist:** conceptualization (equal), data curation (equal), formal analysis (equal), investigation (equal), writing – original draft (equal). **Rasmus Mohr Mortensen:** formal analysis (equal), methodology (equal), writing – review and editing (equal). **Thomas Bregnballe:** conceptualization (equal), investigation (equal), project administration (equal), writing – review and editing (equal). **Martin Mayer:** formal analysis (equal), methodology (equal), visualization (equal), writing – original draft (equal).

## Conflicts of Interest

The authors declare no conflicts of interest.

## Data Availability

The data that support the findings of this study are openly available on the online repository figshare (Mayer et al. 2024. Location data of European hares from Langli Island, figshare, dataset, https://doi.org/10.6084/m9.figshare.25540243).
